# Dexamethasone intravitreal implant in retinal vein occlusion: real-life data from a prospective, multicenter clinical trial

**DOI:** 10.1007/s00417-016-3431-x

**Published:** 2016-07-26

**Authors:** Nicole Eter, Andreas Mohr, Joachim Wachtlin, Nicolas Feltgen, Andrew Shirlaw, Richard Leaback, For the German Ozurdex in RVO Real World Study Group, Ali Asadi, Elisabeth Bator-Banasik, Peyman Bayati, Erik Beeke, Andreas K. Cordes, Hita Tushar Dave, Ahmed Elshinnawi, Katrin Engelmann, Beatrix Entenmann, Nicole Eter, Christian Foja, Philipp Franko Zeitz, Anna Gross, Rainer Guthoff, Helmut Höh, Riad Khaireddin, Ulf Kretschmann, Florian Kretz, Anja Liekfeld, Andreas Mohr, Andreas Neugebauer, Björn Padge, Uwe Reinking, Bernd Schade, David Schell, Jens Schrecker, Claudia Schuart, Annegret Schumacher, Pankaj Singh, Joachim Wachtlin, Julia Wrede

**Affiliations:** 1 Department of Ophthalmology, University of Münster Medical School, Domagkstr 15, D-48149 Münster, Germany; 2 Eye Hospital St. Joseph-Stift, Bremen, Germany; 3 Augenabteilung, Sankt Gertrauden Krankenhaus, Berlin, Germany; 4 University Eye Care Hospital, Göttingen, Germany; 5 Allergan plc, Marlow, UK

**Keywords:** Branch retinal vein occlusion, Central retinal thickness, Central retinal vein occlusion, Dexamethasone, Intravitreal, Macular edema

## Abstract

**Purpose:**

To evaluate the relationship between duration of macular edema associated with retinal vein occlusion (RVO) and the achievement of vision gain in patients receiving dexamethasone intravitreal implant (DEX implant) in real-world clinical practice, and to define patterns of use of DEX implant and its efficacy and safety in the treatment of patients with RVO in clinical practice.

**Methods:**

This prospective, open-label, multicenter, 6-month observational phase IV study conducted at 70 sites in Germany enrolled patients diagnosed with macular edema following branch or central RVO (BRVO, CRVO) who were given DEX implant. Follow-up visits and evaluations occurred in accordance with normal clinical practice. Re-treatment with DEX implant and use of other RVO therapies was at the discretion of the treating physician. The primary endpoint was mean change in best-corrected visual acuity (BCVA) from baseline at week 12.

**Results:**

The analysis population consisted of 573 patients (64 % BRVO, 36 % CRVO). Patients received a mean of 1.17 DEX implant treatments during the study period; 84.3 % of patients received a single DEX implant and 19.9 % received adjunctive other RVO treatment. Among patients with analyzable BCVA data at baseline and week 12 (*n* = 351), mean change from baseline BCVA at week 12 was −0.16 (standard deviation, 0.30) logMAR (+7.8 approximate Early Treatment Diabetic Retinopathy Study [ETDRS] letters) (*p* < 0.001), and 33.9 % of patients had gained at least 3 lines in BCVA from baseline. Mean change from baseline BCVA at week 12 was +9.5, +7.3, and +5.4 approximate ETDRS letters in patients with macular edema duration < 90 days, from 90 to 180 days, and >180 days respectively. Improvement in BCVA through week 24 and decreases in central retinal thickness were seen in both BRVO and CRVO. The most common adverse drug reaction was increased intraocular pressure. No glaucoma incisional surgeries were required.

**Conclusions:**

DEX implant was effective in improving BCVA and central retinal thickness in patients with BRVO and CRVO in real-world clinical practice. The largest gains in BCVA over 6 months occurred in patients with recent onset macular edema, confirming the benefit of early treatment. DEX implant was well tolerated and had an acceptable safety profile.

## Introduction

Macular edema (ME) after a central or branch retinal vein occlusion (CRVO, BRVO) is a common cause of vision loss [1]. Treatment options include laser in BRVO and intravitreal corticosteroids and antagonists of vascular endothelial growth factor (anti-VEGF) in both BRVO and CRVO [2]. Dexamethasone intravitreal implant 0.7 mg (DEX implant; Ozurdex, Allergan plc, Dublin, Ireland) provides sustained release of the potent corticosteroid dexamethasone [3].

DEX implant was the first medical therapy approved for treatment of ME associated with retinal vein occlusion (RVO). In phase III clinical trials, a single treatment with DEX implant effectively improved best-corrected visual acuity (BCVA) and reduced central retinal thickness (CRT) in patients with ME following RVO [4]. Clinically significant improvements in BCVA and CRT after DEX implant treatment were most likely in patients with ME of shorter duration [5]. In patients who received a second implant after 6 months, re-treatment demonstrated efficacy and safety similar to initial treatment, except for an increase in reports of cataract after the second implant [6]. Cataract and increased intraocular pressure (IOP) are the most common side effects of DEX implant treatment [6, 7].

Randomized clinical trials can provide high-quality evidence of the efficacy of a treatment; these trials typically measure efficacy in carefully selected patient populations with standardization of treatment and follow-up assessments. However, in the population at large, patients are generally less healthy with potentially confounding comorbidities, their use of the treatment of interest may vary, and they may receive other types of treatment as well. Therefore, observational studies of the use of a treatment in clinical practice provide additional valuable information concerning patterns of use and the effectiveness of a treatment in a real-life setting. The results of such studies can be extrapolated to a broader population compared with randomized clinical trials.

The purpose of the present study was to evaluate the relationship between duration of RVO-associated ME and the achievement of vision gain in patients receiving DEX implant in real-world clinical practice. A secondary objective was to define patterns of use of DEX implant and its efficacy and safety in the treatment of patients with RVO in clinical practice.

## Methods

This prospective, open-label, multicenter, 6-month observational study was carried out in Germany from April 2012 through June 2014. The study was conducted in accordance with the tenets of the Declaration of Helsinki and Good Clinical Practice, and was approved by the Landesärztekammer Rheinland–Pfalz Independent Ethics Committee. Informed consent was obtained from all individual participants included in the study. The study is registered with the identifier NCT01571557 at ClinicalTrials.gov.

To be included in the study, patients were required to be ≥18 years of age, diagnosed with ME following RVO, and prescribed DEX implant. Patients were required to provide written informed consent. The only exclusion criterion was previous treatment with DEX implant.

Consecutive patients who met the patient eligibility criteria were enrolled, and DEX implant was administered after evaluations on day 1 (baseline). Follow-up visits and evaluations occurred in accordance with normal clinical practice. Any re-treatment with DEX implant was to be consistent with the Summary of Product Characteristics (SPC) and the treating physician’s normal clinical practice.

Data collected at the baseline visit included demographics, type of RVO, date of onset of ME symptoms, date of RVO diagnosis, ophthalmic history, lens status, and previous therapy for RVO. Efficacy was evaluated by BCVA and CRT on optical coherence tomography (OCT) at baseline and each follow-up visit. CRT could be measured on either time-domain or spectral-domain OCT. The primary endpoint was mean change in BCVA from baseline at week 12. Key secondary efficacy outcome measures were mean change from baseline BCVA at each visit, mean BCVA at each visit, the percentage of patients achieving ≥ 2-line gain in BCVA from baseline at week 12, and mean change from baseline CRT at each visit. Other outcome measures included use of DEX implant injections and other treatments for RVO during the study, use of concomitant ophthalmic medication, ocular surgeries, IOP, and adverse drug reactions (ADRs) (primary safety outcome measure).

Patient and physician satisfaction, tolerability of DEX implant, and continuation of treatment were evaluated at the final visit. The patient and physician were asked to rate their overall satisfaction with the DEX implant treatment as “very good”, “good”, “moderate”, or “insufficient”. The tolerability of DEX implant treatment was rated by both the patient and physician, with possible responses of “very good”, “good”, “moderate”, or “bad”. The physician also was asked whether the patient would receive additional DEX implant injections.

Snellen BCVA was converted to logMAR and to approximate Early Treatment Diabetic Retinopathy Study (approxETDRS) letters for analysis [8]. Visual acuity assessments based on finger count or hand motion were not assigned a Snellen value, and consequently were excluded from analysis. Analysis of change in BCVA or CRT from baseline at a particular visit was based on patients with data at both baseline and that visit. All analyses used observed values (no imputation for missing values) in the analysis population of all patients with no protocol violations. When values were available from multiple visits within a visit window, the value from the visit showing greatest improvement (peak drug effect) was used in the analysis.

Subgroup analysis was performed for patient subgroups defined by diagnosis (BRVO or CRVO), the duration of ME at baseline (<90 days, 90–180 days, or >180 days), treatment for RVO used during the study, previous treatment for RVO, and perfusion status. The duration of ME at baseline was determined using the onset of symptoms (the date when the patient first became aware of a decrease in visual acuity) as the start date of the ME. Due to the observational nature of this study and the variable timing and number of follow-up visits, data from visits within a defined timeframe were grouped for analysis. The windows used for visits were week 6: days 2–63, week 12: days 64–126, and week 24 (month 6): days 127–210. Additional visit windows for patients with data collected for longer than 6 months were week 36: days 211–294, and week 48: day 295–last day in study. These data were not requested by the protocol; however, due to the observational nature of the study, some such data were provided.

Statistical analysis was performed using SAS 9.1.3 or 9.3 software (SAS Institute Inc., Cary, NC, USA) and observed values in the dataset of all available data. The Wilcoxon signed rank test was used to test for changes in mean BCVA and mean CRT from baseline. The planned sample size was approximately 1,000 patients.

## Results

### Study population

A total of 623 patients were enrolled in the study from April 2012 through December 2013. Fifty patients were excluded from analysis due to protocol violations (no DEX implant treatment at the baseline visit, *n* = 32; had been treated previously with DEX implant, *n* = 14; informed consent signed after the initial DEX implant treatment, *n* = 2; or missing documentation of study eye, *n* = 1) or to data errors (*n* = 1). Therefore, the analysis population consisted of 573 patients from 70 sites in Germany.

Five hundred nineteen patients in the analysis population (90.6 %) had at least one documented follow-up visit. Data availability for these patients within the predefined visit windows is shown in Table 1. The study duration (period of data collection and analysis) was more than 24 weeks for some patients, but the typical period of follow-up was 6 months, with 316 patients (60.9 % of patients with at least one follow-up visit) seen within the month-6 visit window. The mean time from baseline to the last follow-up visit with data collection was 155 days (range, 1–656).

**Table 1:** Data availability for the analysis population within visit windows

Visit window^a^	Patients seen within visit vindow (*n*)	Patients with BCVA assessment (*n*)	Mean time until BCVA assessment (days)	Patients with OCT assessment (*n*)	Mean time until OCT assessment (days)
Baseline	573	566		378	
Week 6	436	436	38	172	42
Week 12	380	380	95	198	95
Week 24	316	316	161	158	158
Week 36	87	87	240	37	238
Week 48	26	26	369	11	363

*BCVA* best-corrected visual acuity, *OCT* optical coherence tomography

^a^ Visit windows were baseline: day 1; week 6: days 2–63; week 12: days 64–126; and week 24: days 127–210. Additional visit windows for patients who had data collected beyond the 6 months called for in the study protocol were week 36: days 211–294 and week 48: day 295–last day in study

Baseline characteristics of patients and study eyes in the analysis population are listed in Table 2. Approximately two-thirds of the analysis population (64 %) was diagnosed with BRVO and one-third (36 %) with CRVO. Visual acuity in the study eye ranged from “hand motion” (in 15 patients) and “finger counting” (in 12 patients) to better than 20/20 Snellen. Baseline mean Snellen visual acuity was 0.67 logMAR (approximately 20/100) in the total analysis population, 0.61 logMAR (20/80) in patients with BRVO, and 0.79 logMAR (20/125) in patients with CRVO. Patients with an ophthalmic history of other disease that could affect vision, such as age-related macular degeneration and diabetic retinopathy, were not excluded, and in some cases visual acuity could have been affected by the presence of other macular disease. Approximately 8.2 % (47/573) of patients were reported to have concurrent diabetes mellitus. Fifty-six patients (9.8 %) were diagnosed with glaucoma in the study eye.

**Table 2:** Baseline patient characteristics

Parameter	Total population (*n* = 573)	BRVO (*n* = 367)	CRVO(*n* = 206)
Mean age, years (SD)	72.1 (10.6)	72.1 (10.6)	72.2 (10.4)
Range	35–94	35–94	40–89
Gender, *n* (%)			
Male	287 (50.1)	177 (48.2)	110 (53.4)
Female	286 (49.9)	190 (51.8)	96 (46.6)
Median time since diagnosis, years	0.14	0.14	0.15
Ischemic, *n* (%)			
Yes	126 (22.0)	74 (20.2)	52 (25.2)
No	447 (78.0)	293 (79.8)	154 (74.8)
Median days since onset of ME symptoms	97	98	94.5
< 90 days, *n* (%)	268 (46.8)	170 (46.3)	98 (47.6)
90–180 days, *n* (%)	99 (17.3)	57 (15.5)	42 (20.4)
> 180 days, *n* (%)	206 (36.0)	140 (38.2)	66 (32.0)
Lens status in study eye, *n* (%)			
Phakic	312 (54.5)	189 (51.5)	123 (59.7)
Pseudophakic	42 (7.3)	26 (7.1)	16 (7.8)
Not reported^a^	219 (38.2)	152 (41.4)	67 (32.5)
Glaucoma in study eye, *n* (%)	56 (9.8)	35 (9.5)	21 (10.2)
Mean BCVA, logMAR (SD)^b^	0.67 (0.39)	0.61 (0.36)	0.79 (0.41)
Approximate Snellen	20/100	20/80	20/125
Approximate ETDRS letter score	51	54	45
Mean CRT, μm (SD)	501 (169)	476 (145)	546 (198)
History of previous RVO treatment (study eye), *n* (%)			
Yes	234 (40.8)	147 (40.1)	87 (42.2)
Procedure^c^	110 (19.2)	74 (20.2)	36 (17.5)
Drug-based therapy	215 (37.5)	135 (36.8)	80 (38.8)
Bevacizumab	126 (22.0)	80 (21.8)	46 (22.3)
Ranibizumab	90 (15.7)	56 (15.3)	34 (16.5)
Triamcinolone acetonide	10 (1.7)	2 (0.5)	8 (3.9)
Other	38 (6.6)	22 (6.0)	16 (7.8)
No	319 (55.7)	210 (57.2)	109 (52.9)
Not reported	20 (3.5)	10 (2.7)	10 (4.9)

*BCVA* best-corrected visual acuity, *BRVO* branch retinal vein occlusion, *CRT* central retinal thickness, *CRVO* central retinal vein occlusion, *ETDRS* Early Treatment Diabetic Retinopathy Study, *ME* macular edema, *RVO* retinal vein occlusion, *SD* standard deviation

^a^ In 160 cases, the investigator indicated pseudophakic lens status but did not specify whether the patient was pseudophakic in the study eye, the nonstudy eye, or both eyes

^b^ Twenty-seven patients with best visual function determined as “count fingers” or “hand motion” were excluded from the analysis of BCVA

^c^ Focal laser, pan-retinal photocoagulation, and/or other procedure

The median time since the onset of ME symptoms was approximately 3 months (98 days in BRVO patients and 94.5 days in CRVO patients). The RVO was reported to be ischemic in 22 % of study eyes (20.2 % of eyes with BRVO and 25.2 % of eyes with CRVO). Baseline mean CRT was 501 μm (476 μm in BRVO patients and 546 μm in CRVO patients).

### Treatment

During the study, 668 DEX implant treatments were administered. The mean number of DEX implant treatments per patient was 1.17 in the analysis population, 1.18 in patients with BRVO, and 1.14 in patients with CRVO. The number and timing of DEX implant treatments received by patients is shown in Table 3. The majority of patients (483/573, 84.3 %) received a single DEX implant during the study period. For the 85 patients who received a second DEX implant treatment, the mean time between the first and second implant injections was 155 days (range, 59–378). Five patients who were in the study for much longer than 6 months received a third DEX implant treatment. The mean time between the first and third implant in these patients was 314 days (range, 237–405) (Table 3).

**Table 3:** Number and timing of DEX implant treatments during the study period

Parameter	Total population (*n *= 573)	BRVO (*n * = 367)	CRVO (*n * = 206)
Number of DEX implant injections, no. of patients (%)			
1	483 (84.3)	305 (83.1)	178 (86.4)
2	85 (14.8)	58 (15.8)	27 (13.1)
3	5 (0.9)	4 (1.1)	1 (0.5)
For patients who received second injection (*n* = 90 total):			
Mean days from first to second injection (SD)	155 (47)	151 (42)	164 (56)
Range	59–378	59–337	92–378
For patients who received third injection (*n* = 5 total):			
Mean days from first to third injection (SD)	314 (74)	324 (82)	277 (NA)
Range	237–405	237–405	(NA)
Mean days from second to third injection (SD)	166 (61)	178 (64)	120 (NA)
Range	105–245	105–245	(NA)

*BRVO* branch retinal vein occlusion, *CRVO* central retinal vein occlusion, *DEX implant* dexamethasone intravitreal implant, *NA* not applicable, *SD* standard deviation

Most patients received only DEX implant for treatment of their RVO during the study period, but approximately one in five patients (114/573, 19.9 %) also received other types of RVO treatment, most commonly laser (83/573, 14.5 % of patients) or intravitreal anti-VEGF (26/573, 4.5 % of patients) (Table 4).

**Table 4:** Other treatments for RVO used during the study period

Parameter, *n* (%)	Total population (*n* = 573)	BRVO (*n* = 367)	CRVO (*n* = 206)
Patients who used other RVO treatment in addition to DEX implant	114 (19.9)	57 (15.5)	57 (27.7)
Type of other RVO treatment			
Laser	83 (14.5)	47 (12.8)	36 (17.5)
Panretinal photocoagulation	25 (4.4)	7 (1.9)	18 (8.7)
Focal retinal laser	17 (3.0)	15 (4.1)	2 (1.0)
Grid laser, unspecified, or other	41 (7.2)	25 (6.8)	16 (7.8)
Anti-VEGF	26 (4.5)	12 (3.3)	14 (6.8)
Ranibizumab	14 (2.4)	8 (2.2)	6 (2.9)
Bevacizumab	10 (1.7)	3 (0.8)	7 (3.4)
Aflibercept	2 (0.3)	1 (0.3)	1 (0.5)
Pars plana vitrectomy	4 (0.7)	0 (0)	4 (1.9)
Cryocoagulation	4 (0.7)	0 (0)	4 (1.9)
Other	7 (1.2)	3 (0.8)	4 (1.9)
Unknown	9 (1.6)	3 (0.8)	6 (2.9)

*Anti-VEGF* vascular endothelial growth factor antagonist, *BRVO* branch retinal vein occlusion, *CRVO* central retinal vein occlusion, *DEX implant* dexamethasone intravitreal implant, *RVO* retinal vein occlusion

### Efficacy

#### Visual outcomes

Mean BCVA in the total patient population in each visit window is shown in Table 5. Mean BCVA increased significantly from baseline to week 12 (*p* < 0.001) in patients with analyzable data in both visit windows. The mean change in BCVA from baseline at week 12 (primary endpoint) was −0.16 (standard deviation [SD], 0.30) logMAR (+7.8 approxETDRS letters) (*n* = 351). Among patients with analyzable data in both visit windows, 45.3 % gained at least 2 lines in BCVA and 33.9 % gained at least 3 lines in BCVA from baseline to week 12.

**Table 5:** Mean BCVA in visit windows^a^

	Total analysis population (*n* = 573)	
Visit window^b^	Mean logMAR (SD)	Mean approximate ETDRS letters (SD)
Baseline (*n* = 539)	0.673 (0.387)	51.4 (19.3)
Week 6 (*n* = 418)	0.493 (0.378)	60.3 (18.9)
Week 12 (*n* = 365)	0.522 (0.414)	58.9 (20.7)
Week 24 (*n* = 297)	0.551 (0.426)	57.5 (21.3)
Week 36 (*n* = 81)	0.550 (0.457)	57.5 (22.8)
Week 48 (*n* = 24)	0.538 (0.447)	58.1 (22.3)

*BCVA* best-corrected visual acuity, *ETDRS* Early Treatment Diabetic Retinopathy Study, *SD* standard deviation

^a^ Mean values shown represent the mean for all patients with Snellen visual acuity data within the visit window. Patients with visual acuity measurements based on finger count or hand motion were excluded from the analysis

^b^ Visit windows were baseline: day 1; week 6: days 2–63; week 12: days 64–126; and week 24: days 127–210. Additional visit windows for patients who had data collected beyond the 6 months called for in the study protocol were week 36: days 211–294 and week 48: day 295–last day in study

**Fig. 1:**
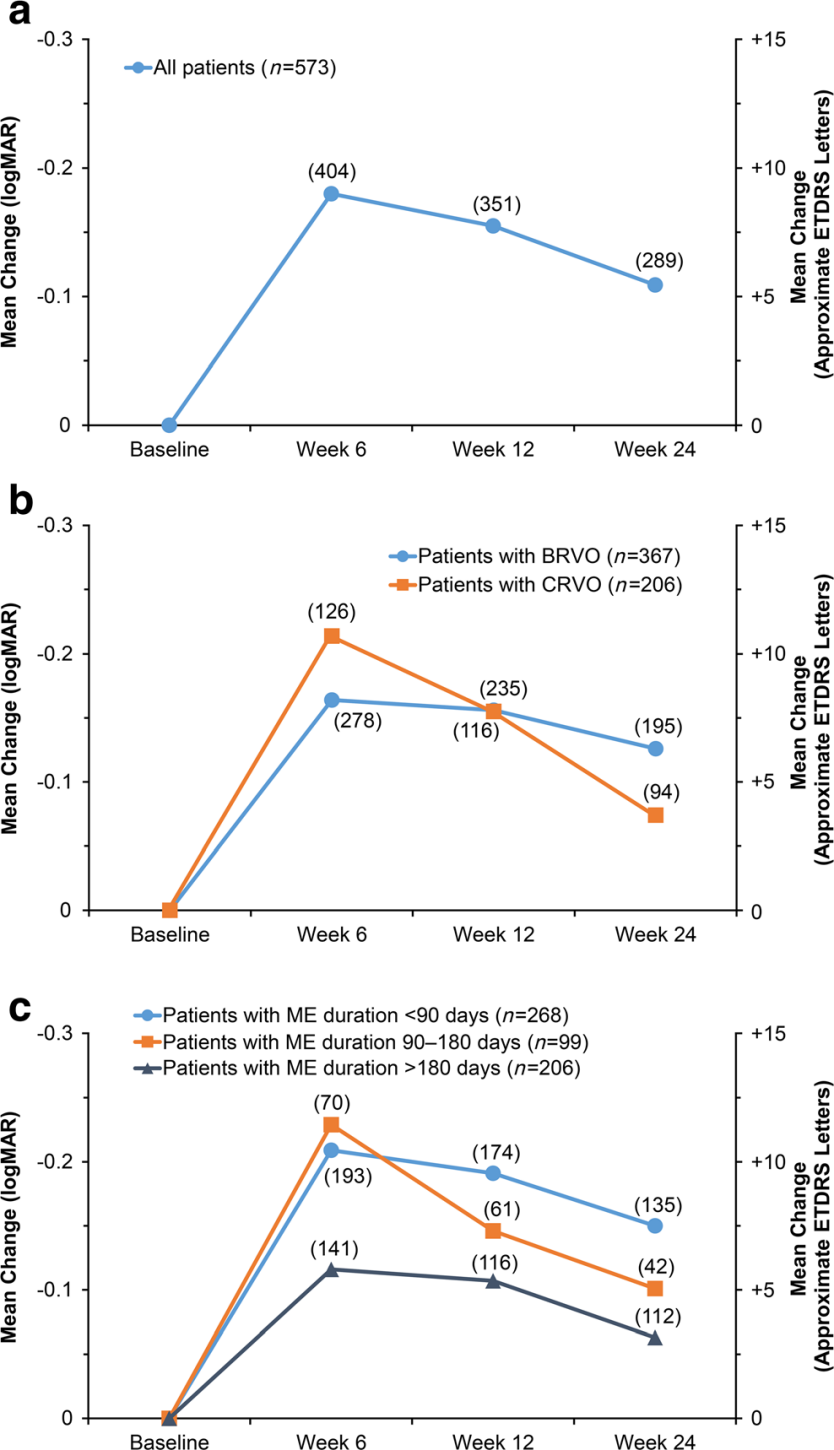
Mean change in best-corrected visual acuity from baseline in (**a**) the total analysis population, (**b**) subgroups diagnosed with BRVO and CRVO, and (**c**) subgroups defined by time since onset of ME symptoms. *Numbers in parentheses* indicate number of patients (*n*). *BRVO* branch retinal vein occlusion, *CRVO* central retinal vein occlusion, *ETDRS* Early Treatment Diabetic Retinopathy Study, *ME* macular edema

Improvement in BCVA from baseline was seen in the total analysis population in each follow-up visit window (weeks 6, 12, and 24) (*p* < 0.0001) (Fig. 1a). The change in BCVA from baseline peaked at week 6 (Fig. 1a). Improvement in BCVA through week 24 was seen in both BRVO and CRVO (Fig. 1b).

**Fig. 2:**
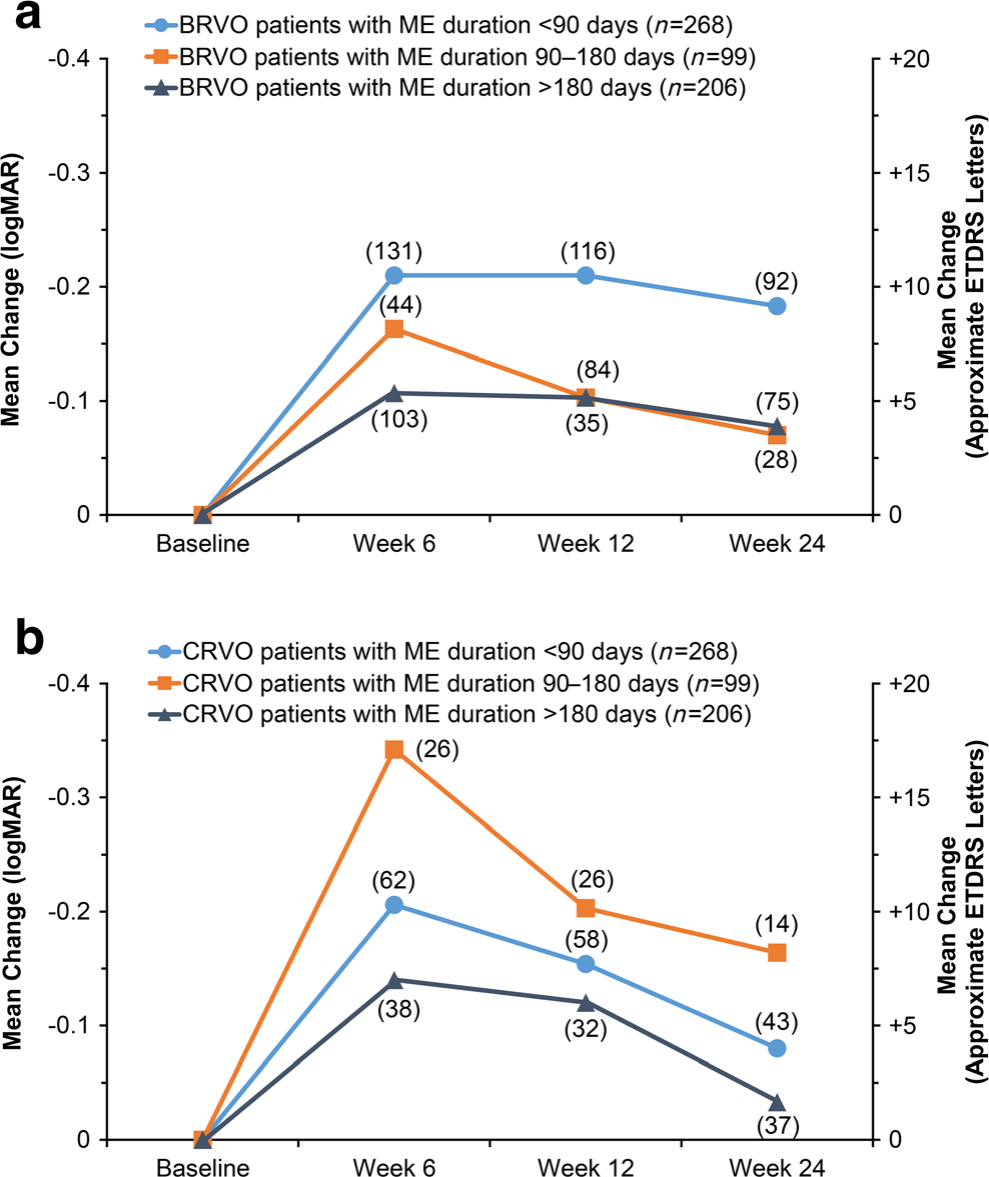
Mean change in best-corrected visual acuity from baseline by time since onset of ME symptoms in (**a**) patients with BRVO and (**b**) patients with CRVO. *Numbers in parentheses* indicate number of patients (*n*). *BRVO* branch retinal vein occlusion, *CRVO* central retinal vein occlusion, *ETDRS* Early Treatment Diabetic Retinopathy Study, *ME* macular edema

A trend for greater gains in BCVA from baseline in patients with ME of more recent onset was evident (Fig. 1c). At week 12, mean change from baseline BCVA was +9.5 approxETDRS letters in patients with ME duration < 90 days (*n* = 174), +7.3 approxETDRS letters in patients with ME duration between 90 and 180 days (*n* = 61), and +5.4 approxETDRS letters in patients with ME duration > 180 days (*n* = 116). In both BRVO and CRVO, there was a trend for better improvement in BCVA when the duration of ME was <90 days versus > 180 days (Fig. 2).

Additional subgroup analysis showed significant improvement in BCVA in patients who received only DEX implant treatment during the study period. The mean (SD) change in BCVA from baseline to week 12 for these patients was −0.160 (0.299) logMAR or +8.0 approxETDRS letters (*n* = 277). Improvement in BCVA in patients who received only DEX implant treatment remained substantial at week 24: the mean (SD) change in BCVA from baseline to week 24 in these patients was −0.120 (0.301) logMAR or +6.0 approxETDRS letters (*n* = 213). This change from baseline BCVA was measured at a mean of 161 days (range, 126–206 days) after the baseline DEX implant treatment.

Improvement in BCVA also was seen in patient subgroups defined by previous treatment and ischemia status. Mean (SD) change from baseline BCVA at week 12 was −0.087 (0.311) logMAR or +4.3 approxETDRS letters (*n* = 134) in patients who had been treated previously with any procedure or medication for RVO in the study eye before study entry, and −0.080 (0.319) logMAR or +4.0 approxETDRS letters (*n* = 105) in patients who had been treated previously with anti-VEGF. In patients who were treatment-naïve, mean (SD) change from baseline BCVA at week 12 was −0.200 (0.286) logMAR or +10.0 approxETDRS letters (*n* = 204). Mean (SD) change from baseline BCVA at week 12 was −0.117 (0.309) logMAR or +5.9 approxETDRS letters (*n* = 75) in patients with ischemic RVO, and −0.166 (0.297) logMAR or +8.3 approxETDRS letters (*n* = 276) in patients with non-ischemic RVO.

A substantial percentage of the patients demonstrated clinically significant improvement in BCVA during the study period. Overall, 47 % (267/573) of patients gained ≥ 2 lines in BCVA from baseline and 36 % (209/573) gained ≥ 3 lines in BCVA from baseline at some point during the study. The mean time to ≥2-line improvement was 58 days (range, 1–312) and the mean time to ≥3-line improvement was 62 days (range, 1–312). These outcomes were almost identical in the subgroups of patients with BRVO versus CRVO. Overall, 48 % of patients with BRVO and 45 % with CRVO gained at least 2 lines in BCVA, with a mean time to ≥2-line improvement of 58 and 59 days respectively, whereas 36 % of patients with BRVO and 37 % with CRVO gained at least 3 lines in BCVA, with a mean time to ≥3-line improvement of 62 and 64 days.

#### Anatomic outcomes

**Fig. 3:**
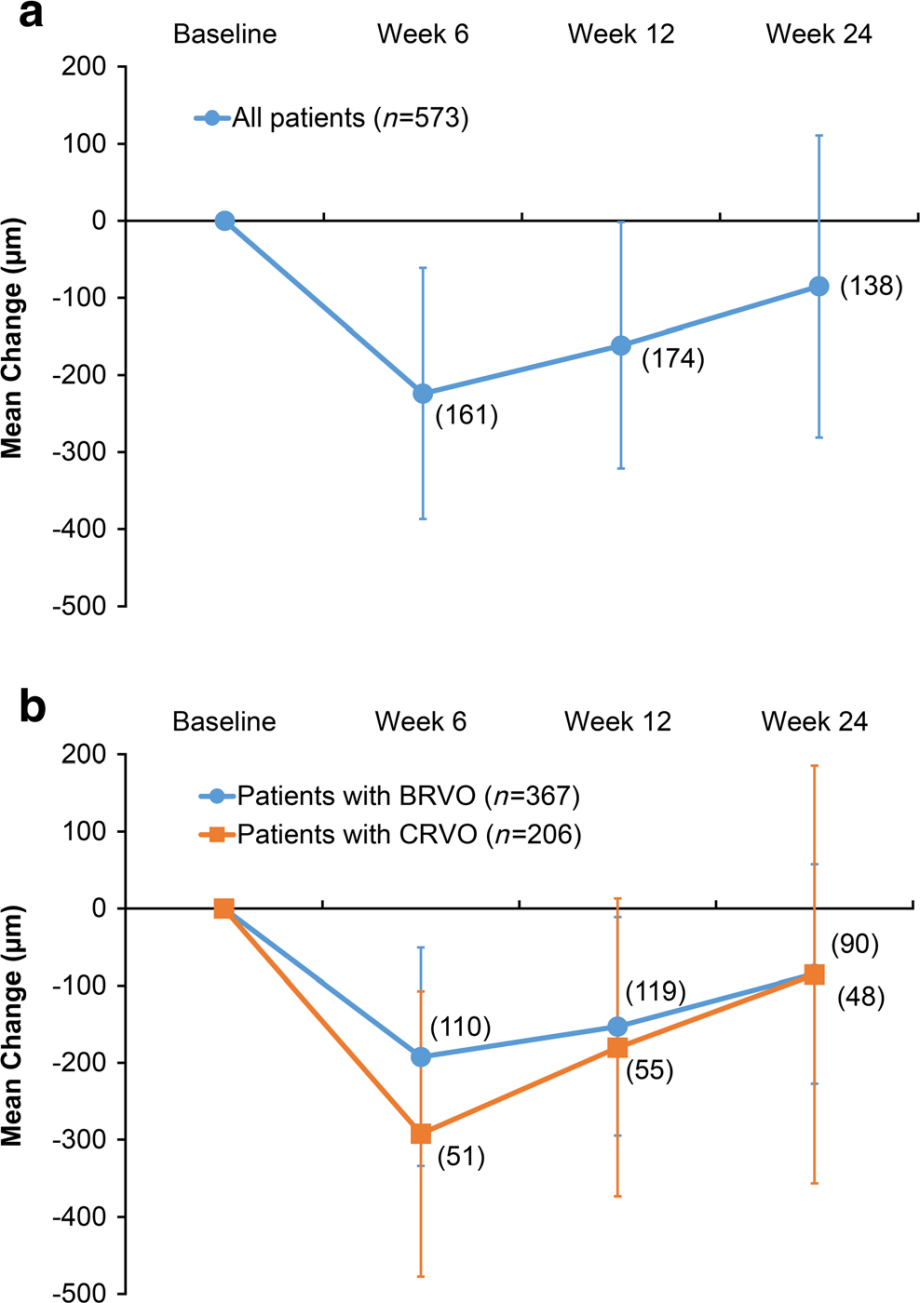
Mean change in central retinal thickness from baseline in (**a**) the total analysis population and (**b**) subgroups diagnosed with BRVO and CRVO. *Error bars* indicate standard deviation. *Numbers in parentheses* indicate number of patients (*n*). *BRVO* branch retinal vein occlusion, *CRVO* central retinal vein occlusion

Three hundred and seventy-eight patients in the analysis population (66.0 %) had an OCT assessment at baseline. Over 90 % (91.1 %, 1047/1149) of the reported CRT measurements at baseline and during follow-up were acquired with spectral-domain OCT, and the remaining measurements were acquired with time-domain OCT. Mean CRT decreased significantly from baseline to week 6 in patients with CRT measurements in both visit windows (*p* < 0.001). The mean decrease in CRT from baseline at week 6 in these patients was >200 μm (Fig. 3a). The peak effect of DEX implant treatment on CRT was at week 6, but mean CRT was also significantly reduced from baseline at weeks 12 and 24 in patients with data in those visit windows (*p* < 0.001). Improvement in CRT was seen in both BRVO and CRVO (Fig. 3b).

### Safety

ADRs were reported in 43 patients (7.5 %). Increased IOP was the only ADR reported in more than two patients; it was reported in 33 patients (5.8 %). There was one serious ADR, deterioration of pre-existing pseudoexfoliation glaucoma necessitating glaucoma laser surgery, in a patient with CRVO. This ADR was rated by the physician as possibly related to treatment.

At the baseline visit, mean IOP before and after the DEX implant injection was 15.4 and 16.2 mmHg, respectively. Seventy-five (13.2 %) of the 570 patients with IOP measurements had IOP > 25 mmHg at some point in the study. Fifty-five (9.6 %) of these patients had IOP >25 mmHg during a single visit window, 13 (2.3 %) had IOP > 25 mmHg during two visit windows, and seven (1.2 %) had IOP > 25 mmHg during three visit windows.

Increases in IOP were most commonly measured during the week-6 visit window and were usually managed with topical IOP-lowering medication. Among the 75 patients with an IOP measurement > 25 mmHg, 21.3 % had IOP > 25 mmHg at the baseline visit, 58.7 % had IOP > 25 mmHg during the week-6 visit window, 38.7 % had IOP > 25 mmHg during the week-12 visit window, and 17.3 % had IOP > 25 mmHg during the week-24 visit window. IOP-lowering medication was used by 7.3 % of patients at baseline and by 16.6 % of patients during the study (Table 6). Among the 75 patients with IOP > 25 mmHg during the study, 13 % used IOP-lowering medication before starting DEX implant, 39 % began use of IOP-lowering medication during the study, and 48 % did not use IOP-lowering medication during the study. No patients had glaucoma incisional surgery during the study period, but nine patients underwent laser trabeculoplasty (Table 6).

**Table 6:** Treatment and procedures for management of IOP during the study period

Treatment or procedure, *n* (%)	Total population (*n* = 573)	BRVO (*n* = 367)	CRVO (*n* = 206)
Glaucoma incisional surgery	0 (0)	0 (0)	0 (0)
Laser trabeculoplasty	9 (1.6)	6 (1.6)	3 (1.5)
Among patients who had laser:			
Baseline use of IOP-lowering medication	1 (11.1)	0 (0)	1 (33.3)
Started IOP-lowering medication during study	1 (11.1)	1 (16.7)	0 (0)
No use of IOP-lowering medication during study	7 (77.8)	5 (83.3)	2 (66.7)
Among all patients:			
IOP-lowering medication used at baseline	42 (7.3)	21 (5.7)	21 (10.2)
IOP-lowering medication used during study	95 (16.6)	52 (14.2)	43 (20.9)

*BRVO* branch retinal vein occlusion, *CRVO* central retinal vein occlusion, *IOP* intraocular pressure

A cataract-related ADR was reported in only one eye (0.3 % of baseline phakic eyes). Nineteen study eyes (6.1 % of baseline phakic study eyes) underwent cataract extraction during the study period.

### Subjective assessments at study end

At the final study visit, physicians rated their satisfaction with DEX implant treatment as “good” or “very good” in 65 % of patients, “moderate” in 19 %, and “insufficient” in 7 % (data were missing for 9 % of patients), and patients rated their satisfaction with DEX implant treatment as “good” or “very good” in 61 % of patients, “moderate” in 22 %, and “insufficient” in 8 % (data were missing for 9 % of patients). Physicians rated the tolerability of DEX implant treatment as “good” or “very good” in 84 % of patients, “moderate” in 6 %, and “insufficient” in 0.2 % (data were missing for the remaining patients). Patient ratings of tolerability were “good” or “very good” in 84 % of patients, “moderate” in 6 %, and “insufficient” in 0.5 % (data were missing for the remaining patients). Among patients with a documented last visit, 53 % were to receive additional DEX implant treatment and 23 % were not to receive additional DEX implant treatment. Data on continuation of treatment were missing for the remaining 24 % of patients.

## Discussion

In this real-life observation study, DEX implant treatment improved BCVA and CRT in patients with BRVO and CRVO. A relationship between duration of ME and treatment efficacy was observed, with larger gains in BCVA over 6 months in patients with recent-onset ME, confirming the benefit of early treatment. DEX implant treatment was well tolerated and had an acceptable safety profile. Increases in IOP that occurred were typically managed with topical medication; however, even among patients with IOP > 25 mmHg, 48 % did not use IOP-lowering medication. No glaucoma incisional surgeries were required during DEX implant treatment.

The mean BCVA gain of +7.8 approxETDRS letters (between 1 and 2 lines in visual acuity) at 12 weeks in this study was similar to the gains in BCVA seen in previous large retrospective studies of real-world use of DEX implant in RVO [9, 10]. DEX implant was effective in patients previously treated with anti-VEGF, consistent with a previous report that nine of ten patients with RVO ineffectively treated with anti-VEGF had improved BCVA and reduced CRT after DEX implant treatment [11]. The relationship between duration of ME and the efficacy of DEX implant treatment seen in this study has also been reported in other studies. In the SOLO retrospective 6-month study, DEX implant treatment was associated with better improvement in CRT in BRVO of shorter duration [12]. Furthermore, in the GENEVA pivotal randomized clinical trials of DEX implant for treatment of RVO-associated ME, shorter duration of ME was associated with better improvement in BCVA and CRT after DEX implant treatment, especially in patients with BRVO [5].

The ADR profile of DEX implant in this study was also consistent with previous reports, and increased IOP was the most frequent adverse effect of treatment. Use of IOP-lowering medication by patients in the study was consistent with medication use in the 6-month GENEVA pivotal trials of DEX implant in patients with RVO-associated ME, in which the percentage of eyes receiving IOP-lowering medication increased from 6 % at baseline to approximately 24 % at 6 months [4], as well as with medication use in a retrospective study of real-world use of DEX implant in Canada, in which 16.7 % of patients with RVO-associated ME who were treated with DEX implant required topical IOP-lowering medication [10]. A slightly higher percentage of patients (29–34 %) were reported to use or begin to use IOP-lowering medications in longer-term retrospective studies of real-world use of two or more DEX implant injections for treatment of RVO-associated ME in Germany and the United States [9, 13]. The incidence of steroid-related cataract progression was very low, probably because of the relatively short study duration of 6 months. Cataract progression is more likely to be reported after multiple implants and a longer duration of treatment [6, 14].

Previous studies have reported the time course of DEX implant effects in RVO. In a prospective study in 19 eyes with RVO-associated ME, improvement in BCVA and CRT was shown to occur rapidly, with significant gains the day after DEX implant treatment [15]. In the GENEVA study, improvement in BCVA was evident by 7 days after treatment, and 3-line gains in visual acuity were maintained for 2–3 months [16]. In this study, mean BCVA improvement peaked at week 6, then declined but remained significant through the month-6 visit window (day 127–210). The most likely explanation for these findings is that for many patients, the optimal time for re-treatment may be <6 months, yet 84.3 % of patients received only one implant treatment during the study period.

When repeat treatment with DEX implant is indicated, the timing of re-treatment varies; however, on average, an interval shorter than 6 months is needed for sustained effectiveness [17–20]. For patients who received a second implant in this study, the mean time between the first and second implant was 155 days (approximately 5 months). This interval is consistent with the interinjection interval of 4.5 months reported in a prospective evaluation of DEX implant in patients with treatment-naïve RVO [21], and with the interinjection interval of 5.6 months reported in the SHASTA study, a large retrospective evaluation in patients with BRVO and CRVO who received at least two DEX implant injections [9]. The mean interval between DEX implant treatments was approximately 5 months (151 days) for patients in the SHASTA study who received no other treatment for RVO-associated ME during the study period [22].

This study had several limitations. Evaluations were at the discretion of the treating physician and according to normal practice, so there was no standardization of assessments (e.g., in how ischemia was determined or CRT was measured), and many patients had missing data, particularly in anatomic measurements. Although OCT provides information useful for guiding treatment decisions in the management of RVO [23], only 66 % of patients had an OCT assessment at baseline, and OCT was not performed regularly during follow-up, presumably because OCT is not reimbursed in Germany. Data on lens status also were missing for over one-third of patients. Visit windows used for analysis were broad to allow analysis of all available data, and on average, BCVA and OCT measurements used for analysis in the week-12 visit window were taken at 13.6 weeks, and those used for analysis in the month-6 visit window were taken at 5.3 months. Although data were collected for 6 months for most patients, some patients had data collected over a longer study period and inclusion of these data affected the analysis of number of treatments received and may have affected the safety findings. Finally, use of other RVO treatments was allowed and may have influenced the efficacy and safety outcomes. However, the majority of patients (80 %) used only DEX implant for treatment of RVO during the study period.

A decrease in the size of the study population over time, due to discontinuations or loss to follow-up, is common in phase IV trials and was seen in this study. Only 316 patients (55.1 % of the analysis population) had available BCVA data within the week-24 visit window. Nonetheless, improvement in BCVA from baseline at week 24 was demonstrated for those patients who had BCVA data at baseline and within the week-24 visit window. Results of the analysis of changes in BCVA and CRT over the course of the study should be interpreted with caution, however, because at different time points, different patients contributed data.

The study populations in randomized controlled trials are typically carefully selected using eligibility criteria. The value of this type of observational study is that the effectiveness of treatment is evaluated in real-world use, where patients have diverse medical histories and comorbidities. The results of this study complement the results of previous randomized controlled trials and demonstrate that DEX implant is effective in reducing CRT and improving BCVA in real-world use.

## Appendix

### Principal investigators in the German Ozurdex in RVO Real World Study Group

Ali Asadi, Augenärzte am Weidenbaumsweg, Hamburg, Germany

Elisabeth Bator-Banasik, Augenärztliche Gemeinschaftspraxis Bator-Banasik, Fischer, Ahaus, Germany

Peyman Bayati, Lichtblick Medizinisches Versorgungszentrum Moers GmbH, Düsseldorf, Germany

Erik Beeke, Klinikum Osnabrück, Osnabrück, Germany

Andreas K. Cordes, Hochkreuz Augenklinik OHG, Bonn, Germany

Hita Tushar Dave, Asklepios Klinik, Hamburg, Germany

Ahmed Elshinnawi, Augenpraxisklinik Lübeck, Lübeck, Germany

Katrin Engelmann, Augenklinik des Klinikum Chemnitz gGmbH, Chemnitz, Germany

Beatrix Entenmann, Augenärzte Waldshut, Waldshut, Germany

Nicole Eter, University of Münster Medical School, Münster, Germany

Christian Foja, Augenärztliche Gemeinschaftspraxis, Leipzig, Germany

Philipp Franko Zeitz, Praxis Zeitz Franko Zeitz Fachärzte für Augenheilkunde, Düsseldorf, Germany

Anna Gross, QAN Gemeinschaftspraxis, Hamburg, Germany

Rainer Guthoff, Klinik für Augenheilkunde Universitätsklinikum Düsseldorf, Düsseldorf, Germany

Helmut Höh, Augenklinik am Dietrich-Bonhoeffer-Klinikum, Neubrandenburg, Germany

Riad Khaireddin, Augen-Diagnostik-Centrum & Augen-Operationen, Bochum, Germany

Ulf Kretschmann, Augen Klinik OWL, Detmold, Germany

Florian Kretz, Universitätsaugenklinik Heidelberg, Heidelberg, Germany

Anja Liekfeld, Klinik für Augenheilkunde, Potsdam, Germany

Andreas Mohr, Eye Hospital St. Joseph-Stift, Bremen, Germany

Andreas Neugebauer, Augenmedizinisches Versorgungszentrum Erfurt, Erfurt, Germany

Björn Padge, Augenärztlichen Gemeinschaftspraxis, Warendorf, Germany

Uwe Reinking, Gemeinschaftspraxis für Augenheilkunde, Salzkotten, Germany

Bernd Schade, Augenärztliche Gemeinschaftspraxis, Besigheim, Germany

David Schell, Augenlaserzentrum Neu-Ulm, Neu-Ulm, Germany

Jens Schrecker, Rudolf Virchow Klinikum Glauchau, Glauchau, Germany

Claudia Schuart, Universitätsaugenklinik Magdeburg, Magdeburg, Germany

Annegret Schumacher, Augenärztliche Gemeinschaftspraxen, Karslruhe, Germany

Pankaj Singh, University Hospital Frankfurt Goethe University, Frankfurt am Main, Germany

Joachim Wachtlin, Augenabteilung, Sankt Gertrauden Krankenhaus, Berlin, Germany

Julia Wrede, Gemeinschaftspraxis, Wiesloch, Germany
